# The Influence of Sagittal Pin Angulation on the Stiffness and Pull-Out Strength of a Monolateral Fixator Construct

**DOI:** 10.3390/bioengineering10080982

**Published:** 2023-08-20

**Authors:** Axel Klemeit, Anna Weber, Christoph Bourauel, Kristian Welle, Christof Burger, Frank A. Schildberg, Christoph Deborre

**Affiliations:** 1 Department of Orthopedics and Trauma Surgery, University Hospital Bonn, 53127 Bonn, Germany; 2 Oral Technology, Dental School, Medical Faculty, University Hospital Bonn, 53111 Bonn, Germany; 3 Clinic for Orthopedics and Trauma Surgery, Bethlehem Health Center Stolberg, 52222 Stolberg, Germany

**Keywords:** biomechanics, monolateral fixator, pin angulation, stiffness, pull-out strength

## Abstract

Monolateral pin-to-bar-clamp fixators are commonly used to stabilize acute extremity injuries. Certain rules regarding frame geometry have been established that affect construct stability. The influence of sagittal pin angulation on construct stiffness and strength has not been investigated. The purpose of this biomechanical study was to demonstrate the effect of a pin angulation in the monolateral fixator using a composite cylinder model. Three groups of composite cylinder models with a fracture gap were loaded with different mounting variants of monolateral pin-to-bar-clamp fixators. In the first group, the pins were set parallel to each other and perpendicular to the specimen. In the second group, both pins were set convergent each in an angle of 15° to the specimen. In the third group, the pins were set each 15° divergent. The strength of the constructions was tested using a mechanical testing machine. This was followed by a cyclic loading test to produce pin loosening. A pull-out test was then performed to evaluate the strength of each construct at the pin–bone interface. Initial stiffness analyses showed that the converging configuration was the stiffest, while the diverging configuration was the least stiff. The parallel mounting showed an intermediate stiffness. There was a significantly higher resistance to pull-out force in the diverging pin configuration compared to the converging pin configuration. There was no significant difference in the pull-out strength of the parallel pins compared to the angled pin pairs. Convergent mounting of pin pairs increases the stiffness of a monolateral fixator, whereas a divergent mounting weakens it. Regarding the strength of the pin–bone interface, the divergent pin configuration appears to provide greater resistance to pull-out force than the convergent one. The results of this pilot study should be important for the doctrine of fixator mounting as well as for fixator component design.

## 1. Introduction

External fixators are widely used in trauma and orthopedic surgery. In its elementary form, a fixator construct consists of bone pins, couplings, and rods placed by the surgeon on a unilateral frame used to stabilize acute severe limb injuries [1,2,3]. For limb reconstruction, deformity correction, osteomyelitis management, and arthrodesis procedures, more complex devices such as the Ilizarov ring fixator are used.

Successful outcomes in both temporary and definitive fracture treatment depend on a stable fixator construct. While axial micromotion in the fracture gap favors callus formation, excessive movement is detrimental and can lead to secondary reduction loss, pin loosening, and malunion [2,4,5,6,7,8,9]. High initial fixator stiffness allows early mobilization and weight-bearing, which in turn promotes fracture healing through micromotion at the fracture site [9,10]. Therefore, understanding the parameters that influence the stiffness of a pin-to-bar-clamp external fixator is fundamental. Both the stiffness of the staked material itself and the dimensions of the pins and rods alter the overall stiffness of the construct [3,11,12,13,14]. As far as the frame geometry is concerned, the stability against axial load and cantilever bending can be increased by a wide pin distribution over a bone segment, a reduction in the pin offset to the fracture gap, a small rod-to-bone distance, a second rod (double stacking), a higher number of pins per segment and, finally, the use of a second pin plane [1,2,3,11,12,14]. In the literature, this additional plane is referred to as the coronal orientation [1,2,15,16,17].

Another quality of a fixator–bone construct is its strength, defined by its ability to maintain integrity against forces. Emphasis is placed on the pin–bone interface because it is the weakest link in the fixator–bone chain and, in addition to pain and loss of reduction, pin loosening appears to promote pin tract infection [3,18,19]. It is known that the primary pull-out strength is influenced by the design of the pins, such as thread pitch and dimensions of the core and thread, as well as their insertion technique, which alters the radial preload [3,20,21,22], while the secondary strength depends on the osseous integration of the pin. The latter can be supported by different pin coatings, such as hydroxyapatite or calcium titanate [23,24].

The main objective of this mechanical study was to investigate the influence of sagittally oriented pin angulation on construct stiffness in a monolateral fixator. Our clinical experience suggests that a configuration with converging pin pairs has higher construct stiffness and a configuration with diverging pin pairs has lower construct stiffness compared to a parallel configuration.

The second objective was to evaluate the strength of the different constructs at the pin–bone interface by pull-out testing. We hypothesized that the pull-out resistance of the angled pin pairs would be higher than that of the parallel version. In addition, a pin loosening condition was to be induced prior to the pull-out test in which the angled pin pairs would show an even greater superiority.

## 2. Materials and Methods

### 2.1. Fracture Model

Hollow short fiber reinforced epoxy cylinders with an outer diameter of 30 mm, a wall thickness of 4.3 mm, and a length of 500 mm (Sawbones, Pacific Research Laboratories Inc., Vashon, WA, USA) were chosen as the bone analog. The goal was to create a basic model comparable to a mid-diaphyseal tibial bone. Cylinders were preferred to anatomical composite tibial bones for more general results and greater accuracy in reproducibility of the fixator–bone constructs. The cylinders were cut into 155 mm segments using a band saw, each representing a fragment of a simulated diaphyseal comminuted fracture gap model.

### 2.2. Frame Assembly

Three types of fixators were created that differed in their pin-pair configuration. The first type had parallel pins, the second type had converging pins with an inter-pin angle of 30°, and the third type had diverging pins with an inter-pin angle of 30° in the sagittal plane. Three constructs of each type were mounted and evaluated for a total of nine specimens.

**Figure 1:**
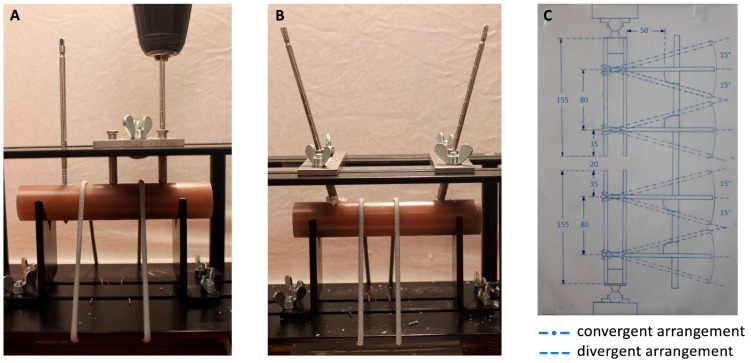
Cylinders loaded into customized jig allowing pin insertion in different pin flights: (**A**) perpendicular pin placement; (**B**) converging pin placement; and (**C**) drawing of the experimental setup as seen sagittally cut through the midsection of the construct. Note the center of rotation of the pins situated in the middle of the bone analog cylinders. The angulated pin arrangements are superimposed with dashed lines. Values in millimeters.

The cylinder segments were loaded with self-drilling half pins (stainless steel Apex Pins, diameter 5 mm, 150 mm × 40 mm; Stryker, Kalamazoo, MI, USA) using a custom-made steel device that allowed either perpendicular or sloped pin insertion at an angle of 15 degrees to the cylinder surface (Figure 1). Because we hoped to aid in subsequent pin loosening, we reduced the radial preload by overdrilling with a 4.5 mm diameter drill bit using a power drill. In addition, a two-way transfixation pin insertion technique was used. The pins were drilled through the cylinders to the end of their thread and drilled back to the desired position pointing out 4 mm from the far cortex. The center of rotation of the angled pins was chosen to be at the center of the cylinder. At this point, the pin span was 80 mm and the pin offset was 35 mm (Figure 1). The pins were reused as long as no apparent plastic deformation had occurred after the pull-out test.

Pin–rod couplings and carbon connecting rods (8 mm × 350 mm) from a previously clinically used Hoffman II external fixation system (Stryker, Kalamazoo, MI, USA) were set up for a basic monolateral, monorod fixator frame. Frame offset and fracture gap size were held constant at 50 and 20 mm, respectively, according to ASTM F1541-17 using a jig made of multiplex boards. A torque wrench was used to tighten the couplings to a torque of 15 Nm. All fixator parts components, except the deformed pins, were reused throughout the test.

**Figure 2:**
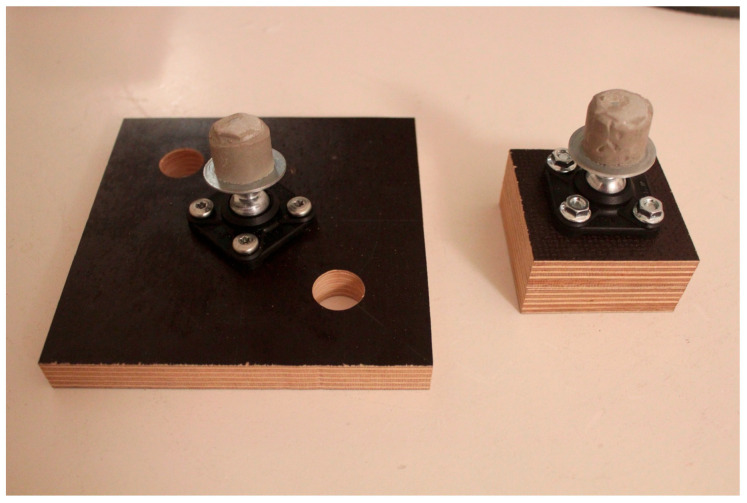
Reusable ball joints (Igubal Flanschlager, Igus GmbH, Cologne, Germany) screwed onto multiplex boards. Note the plaster adjusted to the inner diameter of the composite cylinders.

**Figure 3:**
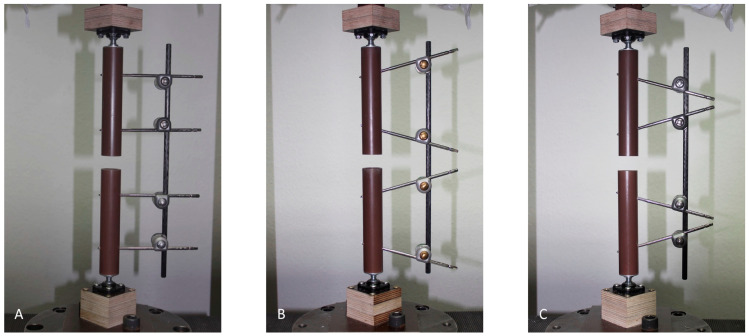
Representatives of the three different test groups loaded into the testing machine: (**A**) parallel pin configuration; (**B**) converging pin configuration; and (**C**) diverging pin configuration.

The bone models were suspended on the testing machine using ball joints (Igubal Flanschlager, Igus GmbH, Cologne, Germany). These suspensions were reused, since their one part was molded of cylindrically shaped plaster adjusted to the inner diameter of the composite cylinders and thus pluggable, while the further part was attached to the testing machine over intercalated multiplex boards (Figure 2). The different assemblies are opposed ready mounted in the testing machine in Figure 3.

### 2.3. Mechanical Testing

All tests were performed on a uniaxial mechanical testing machine (Zwick/Roell Zwick ZmartPro, Ulm, Germany) and were based on ASTM F1541-17. Each specimen was subjected to the following mechanical tests: For a quasi-static axial load test, a peak force of 200 N was chosen because it simulates a partially weight-bearing condition often prescribed for patients with a tibial external fixator. The minimum load was set at 20 N. The load was applied at a rate of 40 N/s. Ten load cycles were performed, the first five being preconditioning cycles for subsequent exclusion. Load and displacement data were recorded by the machine’s transducer for later determination of the corresponding construct stiffness. Next step was a multi-cycle dynamic axial load test of 4000 cycles under load control at 40 N/s. As before, the maximum load was set at 200 N and the minimum load was set at 20 N.

**Figure 4:**
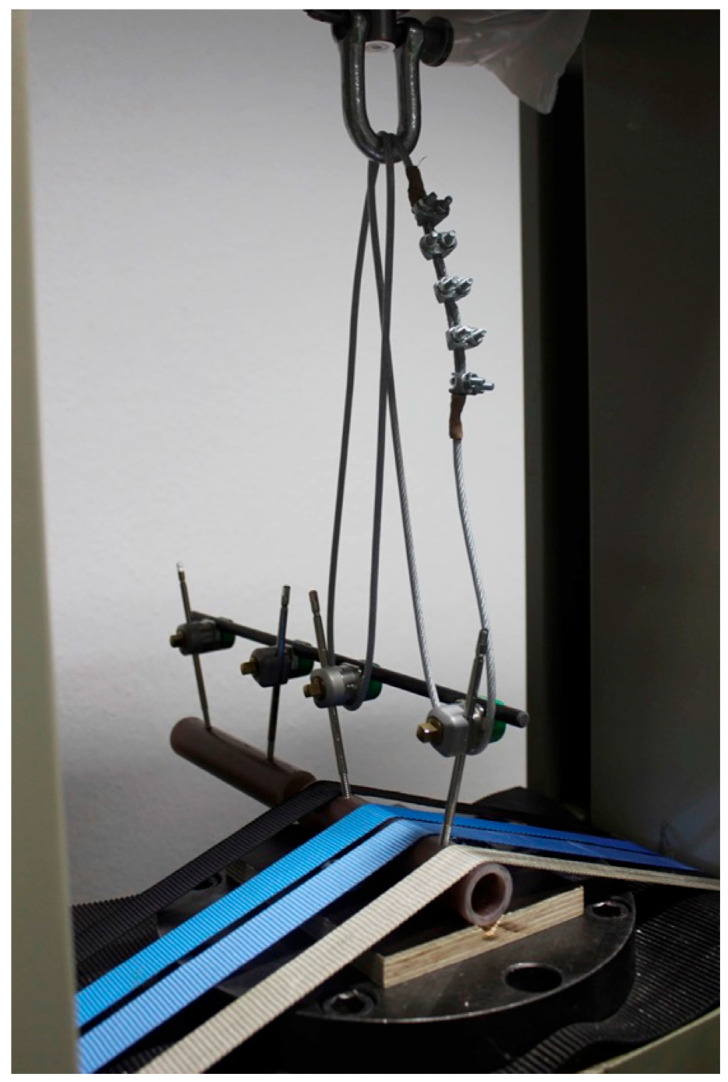
Installation of the pull-out test: The tensile force is exerted through the input platen over cable wires looped around the fixator couplings of one pin pair.

This was followed by a pull-out test of the pin pairs from the cylinder segments. For this purpose, a multiplex board with a cut-out was screwed to the base plate. Each cylinder was placed in the indentation and secured to the base plate with four tension straps, for a total of 18 specimens. A cable wire attached to the input platen was looped around the two corresponding fixator couplings before a tensile force was applied at a speed of 50 mm/min until construct failure (Figure 4). Force and distension were recorded.

### 2.4. Data Analysis

A regression line was calculated from each of the five load–displacement curves taken from the actuator of the testing machine before cyclic loading using Microsoft Excel 365 (Microsoft Corporation, Richmond, VA, USA). The slope of this line represented the mean stiffness of the corresponding specimen. The multicycle load/displacement curves were inspected for irregularities and the constructs were manually examined for signs of damage and pin loosening. The ultimate load at failure was defined as the critical figure of the load/distension curves measured by the transducer of the testing machine resulting from the pull-out test.

### 2.5. Statistics

Statistical analysis was performed using SPSS Statistics version 27 (IBM, Armonk, NY, USA). The mean stiffness of the assemblies was grouped (parallel, converging, and diverging pin configurations) for one-way analysis of variance (ANOVA) followed by a post hoc Tukey test to detect significant differences between groups. The ultimate load at failure during the pull-out test was also grouped for a one-way analysis of variance (ANOVA) and followed by a post hoc Tukey test.

## 3. Results

The purpose of this biomechanical study was to demonstrate the effect of pin angulation in the monolateral fixator using a composite cylinder model. To this end, we established a test system to perform detailed mechanical testing. The resulting model system, as described in the Section 2, represents our first result and could serve as a standardized test system that can be used by the scientific community with a variety of other parameters (implant type, size, etc.).

**Figure 5:**
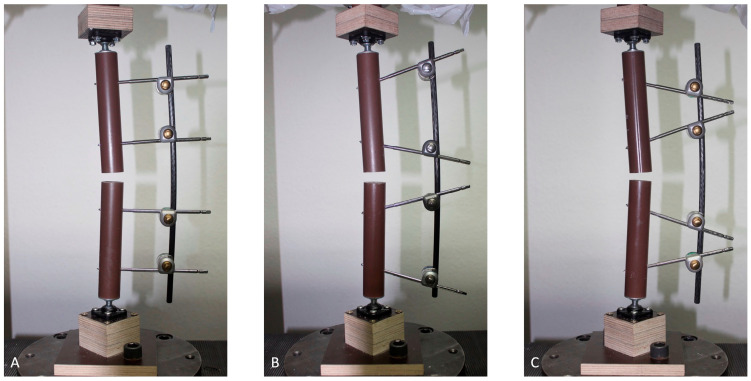
Cantilever bending of the fixator bar under axial load with 200 N: (**A**) parallel pin configuration; (**B**) converging pin configuration; and (**C**) diverging pin configuration.

Visually, there was no pin deformation during axial loading. Cantilever bending of the fixator bar was evident in all settings along the pin flight direction, resulting in irregular fracture gap reduction. The bar bent the highest degree in the divergent pin configuration and the least in the convergent configuration (Figure 5).

**Figure 6:**
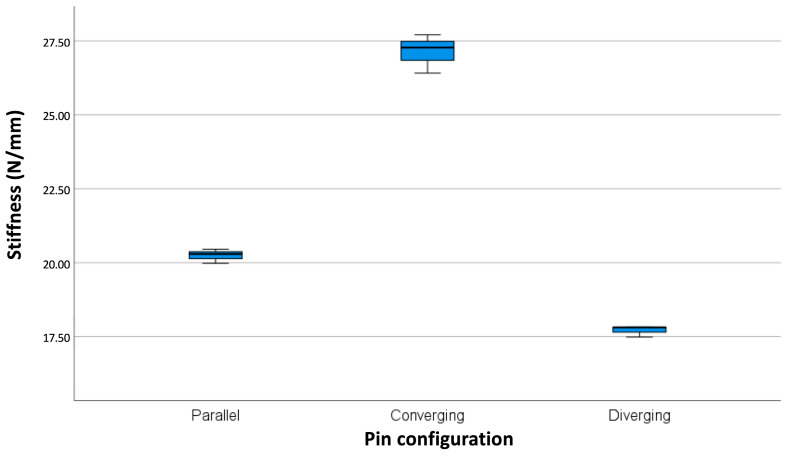
Box plot showing significant differences in stiffness among the different mounting types (*p*-value < 0.01).

There was a significant difference in mean stiffness between the three groups, with the converging configuration being the stiffest (27.13 ± 0.67 N/mm) and the diverging configuration being the least stiff (17.71 ± 0.19 N/mm). The parallel mounting showed an intermediate stiffness (20.24 ± 0.24 N/mm). The results are shown in Table 1 and Figure 6.

**Figure 7:**
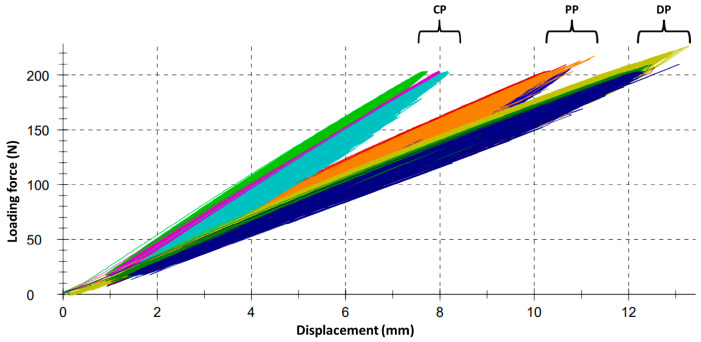
Load/displacement curves as recorded by the testing machine during multi-cycle dynamic axial load test with 4000 cycles. Note the grouping of the curves reflecting the difference in stability of the three pin configurations. Gross irregularities suggesting a loosening or damage of construct parts cannot be stated. CP = converging pins; PP = parallel pins; DP = diverging pins.

There were no obvious signs of fatigue failure in any of the specimens during cyclic axial loading, which would have shown up as gross irregularities in the graphs (Figure 7). In no case was there any evidence of pin loosening by manual inspection.

**Figure 8:**
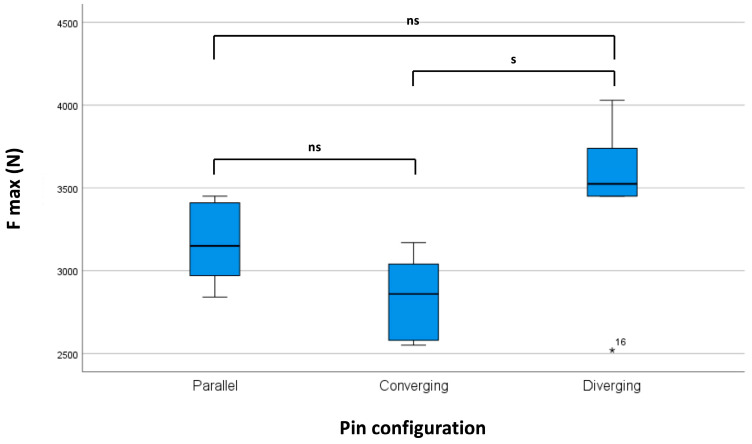
Box plot of the maximum force at failure during extraction of the pin pairs out of the bone analogs. S indicates significant difference (*p*-value < 0.05), ns indicates no significant difference, * ndicates an outlier.

The mean ultimate load at failure of the diverging pin configuration was significantly higher (3.47 ± 0.51 kN) than that of the converging pin configuration (2.84 ± 0.25 kN). A significant difference between the pull-out strength of the parallel pins (3.16 ± 0.24 kN) and other pins could not be confirmed. These results are shown in Table 2 and Figure 8.

## 4. Discussion

A negative correlation between excessive axial and shear motion on the fracture side and early fracture healing has been demonstrated in numerous studies [5,6,7,8,9,25,26]. The stiffness of different fixator designs has therefore played a central role in biomechanical and finite element analyses.

There have been numerous biomechanical investigations of the stability of novel external fixation designs compared to traditional monolateral constructs [27,28,29,30,31,32]. Externalized locking plates and plate-type external fixators have undergone stability and strength testing, as they offer comparable performance to the traditional monolateral fixator, but seem to provide more comfort to the patient [29,30,33]. Another avenue of investigation has been the biomechanical performance of low-cost external fixation devices [32].

Previous biomechanical studies have focused on the design of the basic monolateral external fixators [11,12,15]. As mentioned above, certain rules have been established that contribute to a stable construct. Modern finite element analysis agrees with these findings, but challenges the “near and far” rule of pin placement [34].

Oni et al. have biomechanically demonstrated the beneficial properties of pin angle offset in the transversal plane on fixator stability in a Shearer-type fixator [15]. In addition, Egan et al. calculated a significant gain in torsional stiffness and a “reduction in maximum resultant translation” of the fracture gap under bending loading with increased angular pin separation using a finite element approach [16]. The focus was on pin deflection, as the fixator bar was found to be inflexible.

While the effect of angular pin separation in the sagittal plane on the stiffness of ring-type external fixators has been biomechanically investigated with positive results [4], there has not been a single study addressing the influence of sagittally angled pins in a monolateral fixator.

As we suspected, such a converging pin configuration resulted in increased stiffness under axial loading compared to a parallel pin configuration. There was a 34% increase from a mean of 20.24 N/mm to a mean of 27.13 N/mm when the pin pairs were convergently angled. In contrast, the stiffness decreased by 13% to a mean of 17.71 N/mm when the pin pairs were divergent. The effect is even more pronounced when comparing the converging configuration to the diverging configuration: It was 53% stiffer on average.

The absolute stiffness values are at the lower end of the very wide range of data previously found for monolateral fixators under axial loads ranging from 0.13 to 1157.80 N/mm [12,13,15,29,30]. Compared to a mounting offering a stability at the upper end of the range, as reported in the study by Shi et al. [29], the lower caliber of the bar with 8 mm, a higher frame offset of 50 mm (as recommended by ASTM), and two pins only on either side of the fracture gap explain our clearly lower rigidity. Another factor may be the choice of ball-joint suspensions in our setup compared to a rigid suspension in other studies [12,29,30,31,32]. Since we kept all parameters expecting the pin angulation constant, our relative results between each group should serve well for a general conclusion.

Cantilever bending of the fixator bar under axial load was evident in all specimens. Contrary to the literature [12,16,35], the pins were not the weakest component of our constructs. This may be explained by the relatively small size of the 8 mm bar compared to the 5 mm pins. The gain in stability with a converging configuration seemed to be a matter of increasing the pin span and, thus, decreasing the pin offset to the fracture gap, resulting in a shorter distance of the free bar section bridging the fracture gap. This shorter bar section corresponded to a shorter lever arm, which increased the rigidity against bending forces.

It is likely that increasing the frame offset and using a thicker or second bar would change the proportions in such a way that the pin stress would become more pronounced. We believe that such a condition would still favor the converging pin configuration. The bone fixator construct shown with its opposed angled pin arrangement, is reminiscent of a truss and would presumably best absorb the bending moment occurring in the pin flight direction under axial compression.

In the case of parallel and convergent pin mounting, the asymmetric couplings were installed on the inside of the pin pairs. Due to lack of space between the pins in the diverging setup, the fixator couplings were placed on the outside of the pin pairs. If the couplings were mounted in the same way as in the parallel and convergent constellations, an even greater effect towards loss of stability can be expected.

It has been suggested that the ideal positioning of a monolateral fixator on the tibia is with its pins set in anterior–posterior (AP) flight, as this is the best way to control the predominant bending moment that occurs in AP direction both under early mobilization and progressive weight-bearing [15,16,36]. Although we did not perform a bending test, our model had the freedom to bend in all three dimensions through the installation of ball-joint suspensions at both ends of the model, as suggested by ASTM. Visually, cantilever bending of the fixator bar in the direction of pin flight was evident, resulting in asymmetric fracture gap reduction, with the gap being the smallest on the opposite side of the fixator. The difference in gap reduction between the different groups was also evident and can be seen in Figure 6. It is therefore reasonable to assume that the convergent pin configuration would also have superior bending stiffness in the sagittal plane compared to the parallel and divergent versions. Although other studies included bending loading tests, they lacked the important feature of a ball-joint suspension in their axial loading mode by firmly embedding the ends of the bones or bone analogs and thus locking them to the base and loading plate [12,29,31,32].

The manipulation of the pin–bone interface by overdrilling 0.5 mm beyond the core diameter of the half-pins and the use of a two-way insertion technique must be critically mentioned. This procedure was chosen because a pretest without manipulation of the pin–bone interface did not show the hoped-for pin loosening after cyclic loading. The manipulation of the pin–bone interface could be expected to affect the primary stiffness of the constructs. No palpable loosening of the pins was observed either before or after cyclic loading. Even if the condition had an influence on the stiffness of the constructs, it was the same for all groups and thus not tangential to the relative stiffness.

No fatigue failure occurred in either configuration during 4000 cycles of cyclic axial loading. We had postulated a superior pull-out strength of angled pin pairs over parallel pairs. Motivated through an assumed cant effect of angled pins independent of their threading into the bone, we aimed to induce pin loosening. This should have served as an amplifier of a supposed advantage in pull-out strength. The desired pin loosening was not achieved. Therefore, only the pull-out strength of a sound pin–bone interface was analyzed.

Pin loosening relies upon adverse cortical bone remodeling and bone thread resorption as a reaction to shear forces at the pin–bone interface [18,37]. In addition to the histopathologic response, which was lacking in our model, the material density and wall thickness of the composite cylinders may explain the similar strength of all configurations. The firm grip of the pin threads in the bone analog may have pushed the expected cant effect into the background. Further investigation with a more delicate bone analog, such as thin-walled composite cylinders filled with solid foam to simulate metaphyseal or osteoporotic bone, would be a reasonable next step.

There was a significant difference in pull-out strength between the diverging and parallel configurations, favoring the diverging configuration. This result could be explained by the possibility of a more even distribution of the tensile force exerted on the two fixator clamps by the cable wire looped around them. Because the clamps were closer together in the diverging configuration, the tensile force vectors of the cable were less angled to each other than in the parallel and even less than in the converging arrangement. Presumably, this resulted in a less pronounced preference of one pin over the other at the moment of pin slippage.

A correlation between the stiffness of a fixator–bone construct and its durability at the pin–bone site has been suggested [18,36]. When using a monolateral fixator as definitive fracture treatment, stiffness becomes essential. By avoiding excessive motion at the fracture site, a stiffer construct trends to result in earlier and more reliable callus formation. The callus itself absorbs the forces acting on the fracture side, relieving the pins and reducing stress at the pin–bone interface [38]. Based on our results, a converging pin configuration would be expected to provide better durability with a lower risk of pin loosening compared to a parallel one. Conversely, a divergent pin configuration would increase the risk of pin loosening.

This study has several limitations. Due to reduced technical feasibility, cantilever and torsional testing were omitted and only axial loading experiments were performed. Because this loading mode best simulates the hypothesized predominantly compressive physiologic loading at the fracture site during weight-bearing [15,39,40], and because we allowed the bone-fixator construct freedom of movement in all three dimensions, we believe that this model is still well suited to describe the clinical relevance to the lower limb.

Another weakness of this study is the use of previously clinically used fixator components. In addition, the material was reused throughout the different tests as long as no obvious plastic deformation had occurred.

In addition to the aforementioned attempt to induce pin loosening in vitro, the question of the transferability of our pin-pair extraction technique to clinical reality remains. Applying equal tensile forces to two adjacent pins is unlikely to occur in vivo. The result of such an extraction test is highly dependent on the experimental setup, which allows for more or less distribution of the tensile force on the pins. Measuring and comparing the insertion and extraction forces of each pin, as performed by Pettine et al., and subsequent studies investigating the effect of pin coating with hydroxyapatite or calcium titanate [18,23,24], would be one way to overcome this problem.

The main strength of this study is its simple design. It has the character of a pilot study and, since its design conforms to ASTM, it is easy to reproduce with the possibility of modifying other parameters.

The advantages of a stiffer construct as provided by a converging pin configuration are well known: better ability to maintain primary reduction of a fracture, better patient comfort due to less motion and therefore pain at the fracture level, higher chance of fracture healing, lower risk of pin loosening and, therefore, pin tract infection. All of this would be possible without sacrificing any of the benefits of the pin-to-bar-clamp external fixator. Compared to more dimensional or ring fixators, it is easier and faster to apply, limits the risk of iatrogenic soft tissue and nerve/vascular damage, and is less bulky and therefore more comfortable for the patient. However, due to its limited stiffness, it is rarely used for definitive fracture treatment. In locations where more dimensional or transfixation constructs are problematic due to limited pin corridors and conflicts with free extremity motion, as in the femur and upper extremity, this mounting option should be of great interest. In the case of fractures extending to the metaphyseal zone, joint integration by attaching a frame to the adjacent bone could be avoided with this method. In the femur, a forced wide pin offset could be counteracted by this pin angulation technique. Overall, the surgeon should be aware of this feature. However, to be clear, this study needs to be further validated and does not yet allow for definitive conclusions to actually change external fixation protocols in the clinic. In addition, our findings may open up new areas for product development, such as drill guides, multi-pin clamps, or entire scaffolds.

In contrast, the negative effect of a divergent pin cluster arrangement on axial stiffness could be demonstrated. Knowing the weakening effect, this mounting option should be chosen with caution, as in the case of forced adaptation to soft tissue injuries.

This is the first attempt to show the biomechanical effect of sagittally oriented pin angulation in a monolateral fixator. The mechanical behavior of constructs with further modified parameters under different loading modes needs to be evaluated in future studies. An alternative approach could be finite element analysis, which offers the possibility of targeting specific parameters with less costly and time-consuming methods.

## Figures and Tables

**Table 1:** Descriptive statistics of the axial stiffness of the different pin configurations (parallel, converging, diverging); SD: standard deviation.

Pin Configuration	Sample	Stiffness (N/mm)	Mean Stiffness ± SD (N/mm)
Parallel	1	20.29	20.24 ± 0.24
	2	20.46	
	3	19.98	
Converging	4	27.71	27.13 ± 0.67
	5	26.41	
	6	27.27	
Diverging	7	17.82	17.71 ± 0.19
	8	17.49	
	9	17.81	

**Table 2:** Descriptive statistics of the ultimate force at failure resulting from pull-out testing using the different pin configurations (parallel, converging, diverging); SD: standard deviation.

Pin Configuration	Sample	F Max (kN)	Mean F Max ± SD (kN)
Parallel	1a	3.45	3.16 ± 0.24
	1b	2.84	
	2a	3.16	
	2b	3.14	
	3a	2.97	
	3b	3.41	
Converging	4a	2.87	2.84 ± 0.25
	4b	3.04	
	5a	2.85	
	5b	2.55	
	6a	3.17	
	6b	2.58	
Diverging	7a	3.53	3.47 ± 0.51
	7b	4.03	
	8a	3.52	
	8b	2.52	
	9a	3.45	
	9b	3.74	

## Data Availability

Not applicable.
